# Non-thermal plasma disinfecting procedure is harmless to delicate items of everyday use

**DOI:** 10.1038/s41598-023-42405-6

**Published:** 2023-09-19

**Authors:** V. Scholtz, J. Jirešová, L. Fišer, K. Obrová, M. Sláma, M. Klenivskyi, J. Khun, E. Vaňková

**Affiliations:** 1 Department of Physics and Measurements, University of Chemistry and Technology, Prague, Czech Republic; 2 Division Molecular Microbiology, St. Anna Children’s Cancer Research Institute (CCRI), Vienna, Austria; 3 Faculty of Science, University of Hradec Kralove, Hradec Králové, Czech Republic; 4 Department of Biotechnology, University of Chemistry and Technology, Prague, Czech Republic

**Keywords:** Plasma physics, Electronic devices, Antiviral agents, Electronic materials

## Abstract

Non-thermal plasma (NTP) is a well-known decontamination tool applicable for a wide range of microorganisms and viruses. Since the recent COVID-19 pandemic highlighted the need to decontaminate all daily used items, it is highly desirable to address the applicability of NTP, including its possible harmful effects. To the best of our knowledge, a comprehensive characterization of NTP effects on sensitive materials is still lacking. We investigated the potential damage to common materials of daily use inflicted by air atmospheric NTP generated in Plasmatico v1.0. The materials tested were paper, various metals, and passive and active electronic components modelling sensitive parts of commonly used small electronic devices. The NTP-exposed paper remained fully usable with only slight changes in its properties, such as whitening, pH change, and degree of polymerization. NTP caused mild oxidation of copper, tinned copper, brass, and a very mild oxidation of stainless steel. However, these changes do not affect the normal functionality of these materials. No significant changes were observed for passive electronic components; active components displayed a very slight shift of the measured values observed for the humidity sensor. In conclusion, NTP can be considered a gentle tool suitable for decontamination of various sensitive materials.

## Introduction

Plasma is a partially or fully ionized gas consisting of ions, electrons, and neutral particles that exhibit collective behavior and quasi-neutrality. In contrast to thermal plasma, in which the temperatures of electrons and gas are close, a non-thermal plasma (NTP) is highly non-equilibrium, i.e., the gas temperature is much lower than the electron one. NTP with the gas temperature as low as the room temperature is usually called a cold plasma and is currently in great demand. The main benefit of this type of plasma is that it does not cause thermal damage to the processed objects, so it may be used to process various thermolabile materials. It does not pollute the surface with chemically aggressive substances, and its investment and operating costs are very low. NTP can be easily generated using different types of electrical discharges in gases. Generation can be simplified by igniting electric discharges directly in the air, where the gas or vacuum technique are not required. This also greatly simplifies the application of the generated plasma to the desired objects. The most common sources of NTP are corona discharge, dielectric barrier discharge, radio frequency or microwave discharges and a range of devices known as plasma jets. A very nice comprehensive introduction to NTP and its generation can be found in an older but still actual review^1^.

NTP exhibits antimicrobial^2–7^ and antiviral effects^8,9^, and its applications have been the subject of intensive research in recent decades^6,10–12^. As NTP is typically generated in ambient air, the largest proportion of microbicidal and virucidal agents are reactive oxygen species (ROS) and reactive nitrogen species (RNS)^13^. Possible described uses of NTP are the decontamination or disinfection of surfaces and liquids^11,14^ or various medical applications such as the treatment of skin or nail infections, where NTP can have, in addition to disinfectant effects, also other beneficial effects on tissues and wound healing^15–17^. Although it is generally accepted that NTP is very gentle in sensitive materials treatment, many studies addressing microbicidal properties do not focus on possible NTP side effects. This topic became of relevance in reported NTP applications for the decontamination of protective equipment during the pandemic of COVID-19. Several studies were published at this time of lack of protective equipment, mainly face masks and respirators^8,9,18–20^. In our previous study Obrová et al.^8^, we reported that NTP can be advantageously used for the decontamination of respiratory P3 R filters without affecting their filtration capacity. Due to the general urgency, these studies, including ours, reported a reliable but simple demonstration of decontamination and reuse of the treated objects, while more complex studies aimed at the modifications and possible damage to the studied materials were carried out only marginally.

Modifications of various material surfaces with NTP have been investigated^21–24^, but most often aimed at specific changes in surface properties rather than addressing potential material damage. Setiawan et al.^21^ reported a mini-review describing the plasma jet-produced NTP treatment of different materials (e.g., polymers, stainless steel, carbon material) and concluded that this treatment modifies the surface of the materials with the desired functional groups, changes its wettability, but does not change the properties within the material. Alavi et al.^25^ demonstrated that an atmospheric pressure plasma jet can locally activate the surfaces of 3D-printed structural polymers for the reagent-free covalent attachment of proteins and hydrogel. This is achieved due to surface oxidation indicated by the increase of oxygen atomic concentration from 0% for the untreated surface to 15% after NTP exposure. These changes are caused by the creation of ether, carbonyl, and carboxylic groups and lead to a change of surface wettability. Similarly, the wettability was improved and the chemical composition of polylactic acid surface was changed using plasma jet^22^ or dielectric barrier discharge^23^.

Other work^26^ reported the successful fungal decontamination of paper materials of cultural heritage by atmospheric discharge with runaway electron plasma. In the discussion, the authors note that degradation of treated materials should be monitored, but they expect that degradation of papers by fungi can be much more intensive than with plasma decontamination. A similar view is presented in Pietrzak et al.^27^. In Skácelová et al.^28^ authors dealt with the modification of paper and paperboard by corona and diffuse coplanar surface barrier discharge. They observed an effective modification of the surface characteristics and surface functions of the paper materials, in particular, an increase of the wettability with partial relaxation on aging for several hours without changing the bulk properties.

Li et al.^24^ reported the oxidation of platinum, a noble metal, resulting in the formation of an ultrathin PtO_2_ film after exposure to NTP generated in O_2_ atmosphere. Cogollo de Cádiz et al.^29^ reported surface oxidation resulting in undesirable degradation of wolfram, stainless steel and nichrome. However, in this study metals served as electrodes in corona discharge for a long time, intensifying the interaction with the active particles. Under these conditions (an extremely long exposure time—up to 400 h—in a relatively high-power plasma), irregular oxidative films formed and resulted in roughening and peeling of the surface due to erosion. This is a very extreme case, which certainly does not correspond to the common application of NTP for the decontamination of sensitive materials. However, under the conditions that are normally sufficient for decontamination, the extent of the sensitive materials damage has not yet been adequately investigated.

NTP generators of gentle types should be considered in case of sensitive materials decontamination. Therefore, the current study employs a DC-driven point-to-ring type corona discharge operating in ambient air at atmospheric pressure. We focused on documenting changes in properties of selected materials, anticipated (or not) to be altered or damaged by NTP treatment. Since data on possible NTP modifications of various sensitive materials are still lacking, we considered various objects of daily use suitable for NTP-mediated decontamination and selected paper documents, keys, and electronic devices for testing. The following materials were selected for testing: paper as a model organic sensitive material, various metals used daily—typically as contacts in electronic devices, gold and platinum as model noble metals, and components of electronic devices. Using various physics methods, we evaluated changes in the properties of the selected materials in order to assess whether exposure to NTP had detrimental consequences for their continued use. No changes in the properties of the materials exposed to NTP were found, which could preclude further use of these materials for everyday activities.

## Materials and methods

This section contains a description of the NTP apparatus and the selected materials of daily use tested using NTP treatment. The prepared samples were exposed to NTP in a plasma apparatus for 0 (unexposed control), 30 and 120 min (exposure times used for decontamination in our previous study^8^ for the respiratory P3 R filters disinfection), and changes in properties were evaluated.

### NTP apparatus and sample treatment

**Figure 1:**
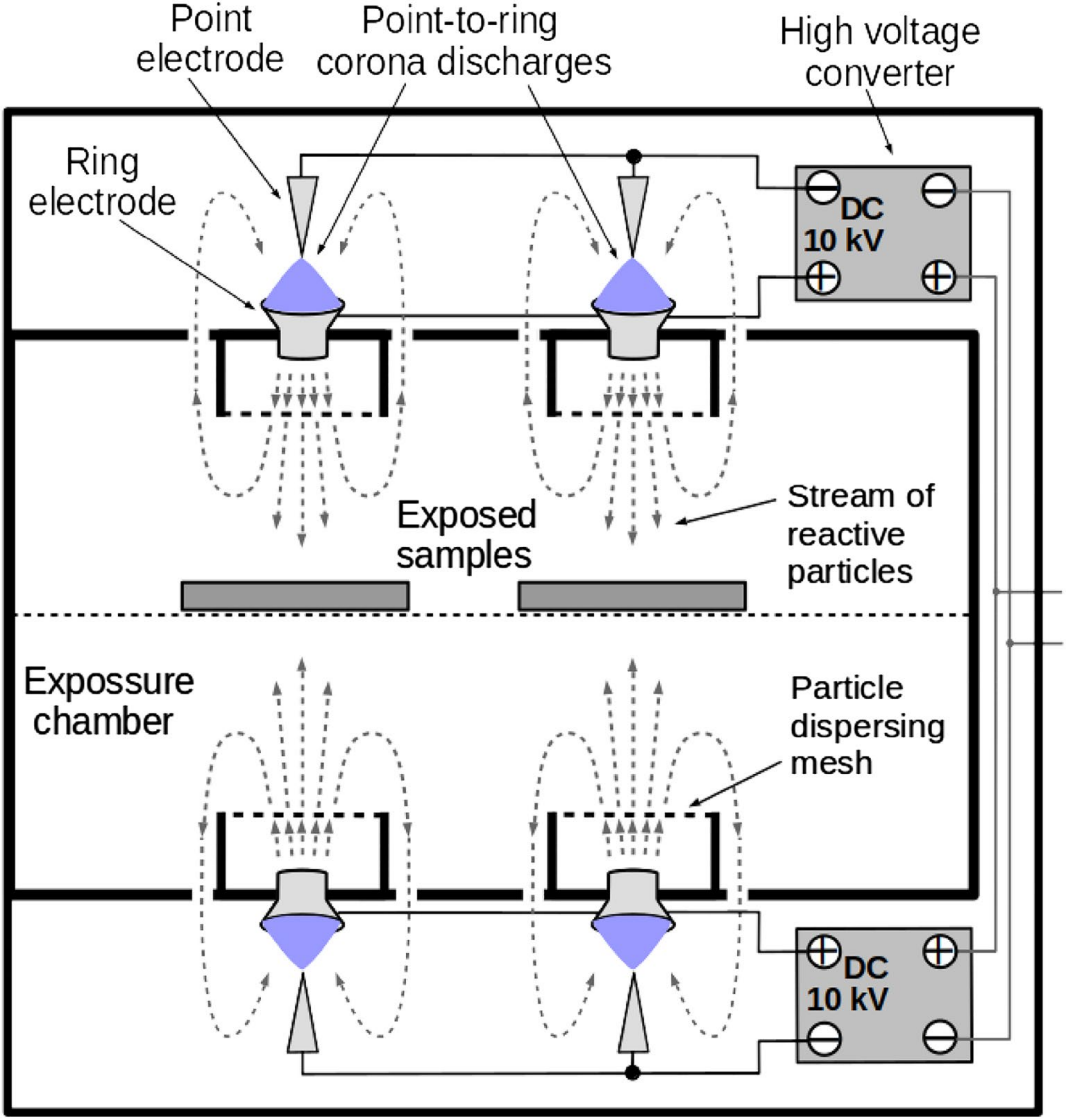
Schematic depiction of the non-thermal plasma (NTP) apparatus. Two matrices of 2 × 2 DC-driven corona discharges of point-to-ring type burn at the bottom and at the top of the chamber; the generated ion wind carries reactive particles through the ring electrode into the space of sample exposure.

NTP was generated by burning eight bipolar DC corona discharges in point-to-ring electrode systems enclosed in an apparatus (Fig. 1) of 17 cm × 18 cm × 18 cm in dimensions. Two matrices of 2 × 2 discharges were placed at the bottom and top of the chamber, respectively. The discharge was addressed in our previous study^30^. Briefly, the corona discharge electrodes were supplied with DC high voltage power supply. The point electrode was a commercially produced stainless steel intramuscular injection needle (Medoject 0.6 mm × 25 mm), oriented perpendicularly to the plane of the ring electrode and connected to the negative pole of the DC high voltage power supply. The ring electrode was a commercially produced brass tube (widening diameter of 8–11 mm), connected to the positive pole of the DC high voltage power supply. In this setup, the negative corona discharge burned close to the point electrode and the positive discharge burned at the circumference of the ring. The distance of the ring electrode from the point one was *d* = 9 mm, the discharge voltage was set to 10 kV and the discharge current corresponded to (50 ± 10) µA. The generated ion wind carried the reactive particles through the ring electrode into the exposure chamber. Exposed samples were placed in the chamber approximately in the middle and exposed for 0 (control), 30 and 120 min.

### Sensitive materials and characterization of their properties after NTP treatment

Paper, metal samples, and electronic components were selected as materials of interest. For paper, an organic material, chemical, optical, and mechanical changes were evaluated upon NTP-produced ROS and RNS interaction, namely: changes in polymerization degree (indicating oxidation-induced breaking of the long polymer cellulose chains), changes in pH (indicating acidification caused primarily by the formation of NO_3_^−^ dissolving to HNO_3_). Optical characteristics were tested as oxidation may cause dye discoloration. Chemical changes in the ratio of selected chemical elements on the paper surface were evaluated by energy-dispersive spectroscopy (EDS). Finally, to determine the overall change in paper quality, mechanical characteristics were addressed.

Metals of various vulnerability [easily oxidizable (copper, brass), oxidizable (stainless steel, aluminum, solder) and noble metals (platinum, gold)] were used. Surface oxidation was anticipated upon NTP treatment of metals; therefore, the EDS was used for analysis and focused on the oxygen-metal ratio.

Electronic components were tested to simulate NTP effects on electronic devices. In this case, open electronic components are more likely to suffer from NTP treatment than completely enclosed ones. Therefore, we included open components in the study. Passive components (resistors and capacitors) of following types were tested: the rather vulnerable small surface mount device (SMD), the bigger and more robust through-hole technology (THT), and resistor trimmers containing open carbon resistance track and track-wiper contact. Passive components were characterized by relevant changes in corresponding electrical quantities: the electrical resistance for resistor and the capacity for capacitor. Active components represent a complex electric circuit, we selected a digital sensor of temperature and humidity, which transmits data via serial bus, and compared the values indicated by sensor to real ones upon NTP-treatment. Although the temperature sensor may also be fully enclosed, the active layer for humidity measurement needs to be open and thus vulnerable.

### Paper

Pieces (190 × 190 mm) of Whatman 1 CHR Chromatography paper (200 × 200 mm, Sigma-Aldrich) were cut and exposed to NTP for 0 (control), 30, and 120 min in six repetitions. Potential inflicted damage was evaluated using the following parameters:

#### Mechanical parameters of NTP-exposed paper

The thickness and grammage of the samples were evaluated with respect to ISO 534 and 536, respectively. Tensile properties such as tensile strength (TS), breaking strength (BS), breaking length (BL) and relative elongation (RE) were measured using the TIRAtest 26005 machine (1993, TIRA Maschinenbau GmbH, Germany) with respect to ISO 1924-2. The paper samples were cut in both directions of their machine direction (MD) and cross direction (CD).

#### Optical parameters of NTP-exposed paper

As representative optical parameters for the surface reflectivity of the measured samples, whiteness and brightness were selected. ISO brightness is defined as the capture of reflected light from a standardized D65 source through a blue filter at a wavelength of 457 nm. This ISO brightness does not entirely correspond to the perception of human eye, as it is less sensitive to the content of optical brighteners in the paper. The CIE whiteness was measured using a standardized D65 source and reflected light was measured over the entire range of visible spectra. This value corresponds to human vision and fully reflects the effect of optical brightening agents. The optical properties of the paper samples were determined using an Elrepho SE 070R (Lorentzen & Wettre).

#### pH value of NTP-exposed paper

Paper samples pH was measured according to ISO 6588-1 as follows: 2 g of sample was extracted in 100 ml of highly pure water for 1 h. The extract pH was measured using the pH 3110 (WTW Wissenschaftlich-Technische Werkstätten GmbH, Germany) at a temperature between 20 and 25 °C.

#### Degree of polymerization of NTP-exposed paper

The degree of polymerization (DP) was determined using a viscosity method published in Milichovsky and Milichovska^31^. Briefly, the paper (cellulose) was dissolved in cadoxen solvent. The viscosity of the resulting solution was measured using an Ubbelohde no. Ia viscosimeter at a concentration of 4 g/l and DP was calculated as follows: DP = 148.3⋅η^1.105^.

#### Energy-dispersive spectroscopy of NTP-exposed paper

Energy-dispersive X-ray spectra (EDS) were recorded by a Quantax 200 spectrometer with an XFlash 6|10 detector (Bruker, Brno, Czech Republic) using 15 kV accelerating voltage of primary electron beam in a TESCAN MIRA 3 LMH scanning electron microscope (TESCAN, Brno, Czech Republic). Paper samples (approximately 5 × 5 mm in size) were mounted on aluminum specimen stubs using a double-sided adhesive carbon tape. The measurements were performed in five repetitions.

### Energy-dispersive spectroscopy of NTP-exposed metal surfaces

Samples of the following metals were tested: copper wire, tinned copper wire (both 0.6 mm thick), brass sheet (thickness 0.5 mm), stainless steel (injection needle 0.7 mm thick), aluminum sheet (thickness 0.075 mm), electric contact solder, platinum wire (0.3 mm thick) and gold wire (0.15 mm thick).

Small pieces (approximately 10 × 10 mm sheets or 10 mm long wires) were exposed to NTP for 0, 30, and 120 min. The surface chemical changes were evaluated by EDS in the same way as described above for paper samples.

### Electronic components

The following passive components were used: SMD resistors R0603(Yageo) *R* = 15 kΩ and capacitors CKS1206 (Yageo) *C* = 56 nF; the THT capacitors TK744 (Tesla Hradec Kralove) *C* = 10 nF and *C* = 15 nF. Components were randomly divided into two groups of six pieces – one group was used as a control, the other was exposed to NTP for 30 and 120 min and electrical properties were measured using a digital multimeter 34461A (Keysight).

Four resistor trimmers TP110 (Tesla Hradec Kralove) of *R* = 10 kΩ with an open carbon resistance track were exposed to NTP and resistance *R*_A_ (between the first terminal and the wiper) and *R*_B_ (between the wiper and the second terminal) were measured before and after the NTP exposure.

Two groups (control and 120 min NTP exposure) of six pieces of the DHT11 digital temperature and humidity sensor (Guangzhou Aosong Electronic Co., Ltd.) were tested for functionality in a cell with constant temperature and humidity after NTP exposure and 14 days later. Afterwards, the sensors were destroyed and the active layers for humidity measurement were evaluated by EDS as described above. The sensor uses a resistance thermometer (range 0 to 50 °C, accuracy of 2 °C) and a vulnerable open active layer using humidity-dependent conductivity (range 20–80%, accuracy of 5%).

## Results

### Changes in paper properties after exposure to NTP

The mechanical parameters determined for 0 (unexposed control), 30 and 120 min NTP-exposed paper (Table S1) showed that, with respect to the calculated deviations, no changes were observed after exposure to NTP.

**Figure 2:**
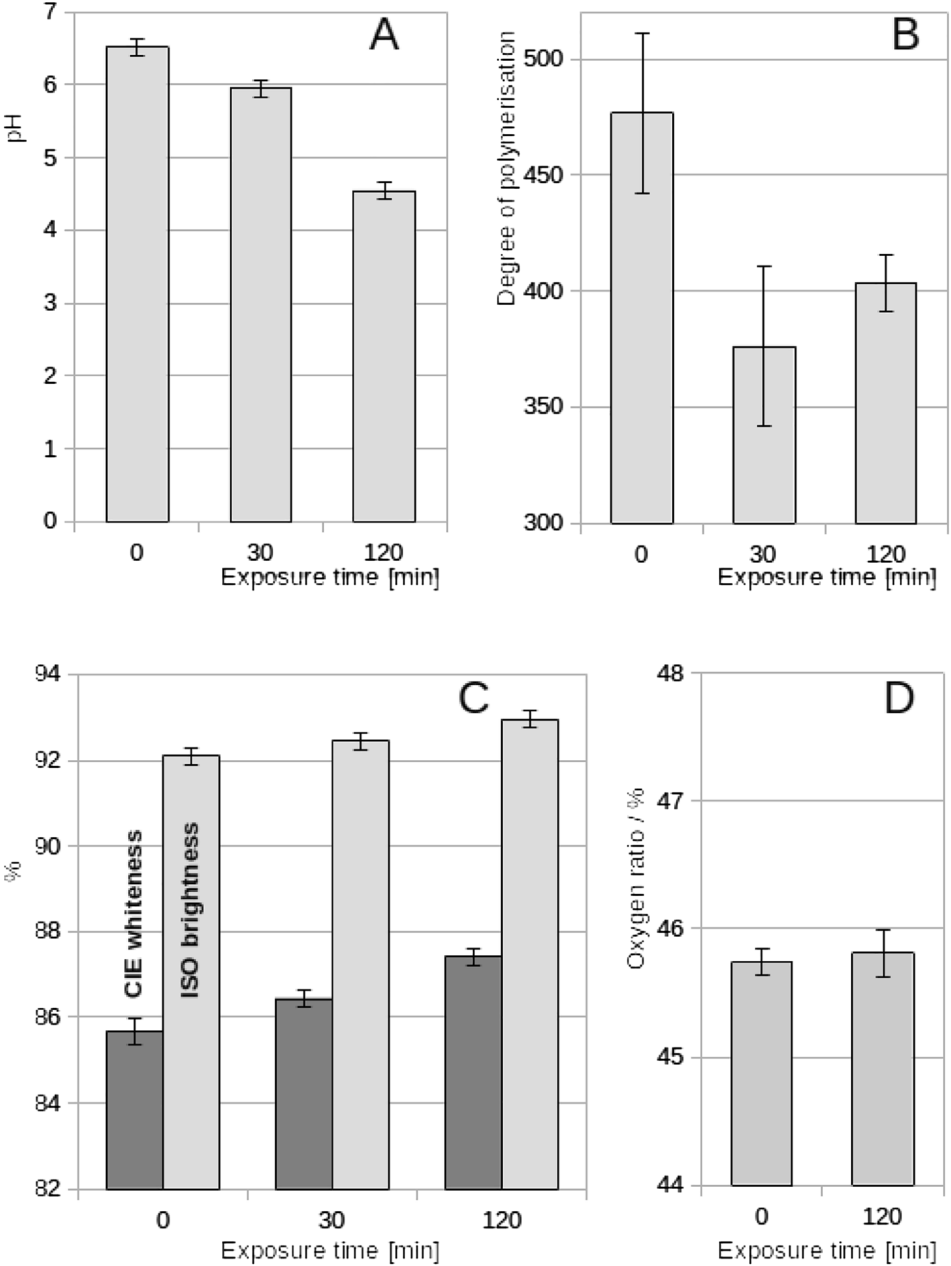
Chemical and optical properties of paper samples after exposure to NTP: (**A**) pH of paper extracts; (**B**) the degree of polymerization; (**C**) optical parameters; (**D**) the oxygen ratio determined by EDS. Results are shown as average of six (**A**,**B**,**D**) or two (**C**) replicates ± standard deviations.

The chemical and optical characteristics of control (0 min exposure) and NTP-exposed (30 and 120 min) paper are shown in Fig. 2. The pH values of the sample extracts decreased from approximately 6.5 to 4.5 (Fig. 2A), indicating acidification of the paper. The degree of polymerization decreased from approximately 475 to 400 (Fig. 2B), indicating a decrease in cellulose fiber length. Both ISO brightness and CIE whiteness increased by approximately 1–3%, indicating a very mild increase in paper reflectivity after 120 min of NTP exposure (Fig. 2C). EDS measurements did not detect changes in the oxygen ratio (Fig. 2D).

### Changes in metal properties after exposure to NTP

**Figure 3:**
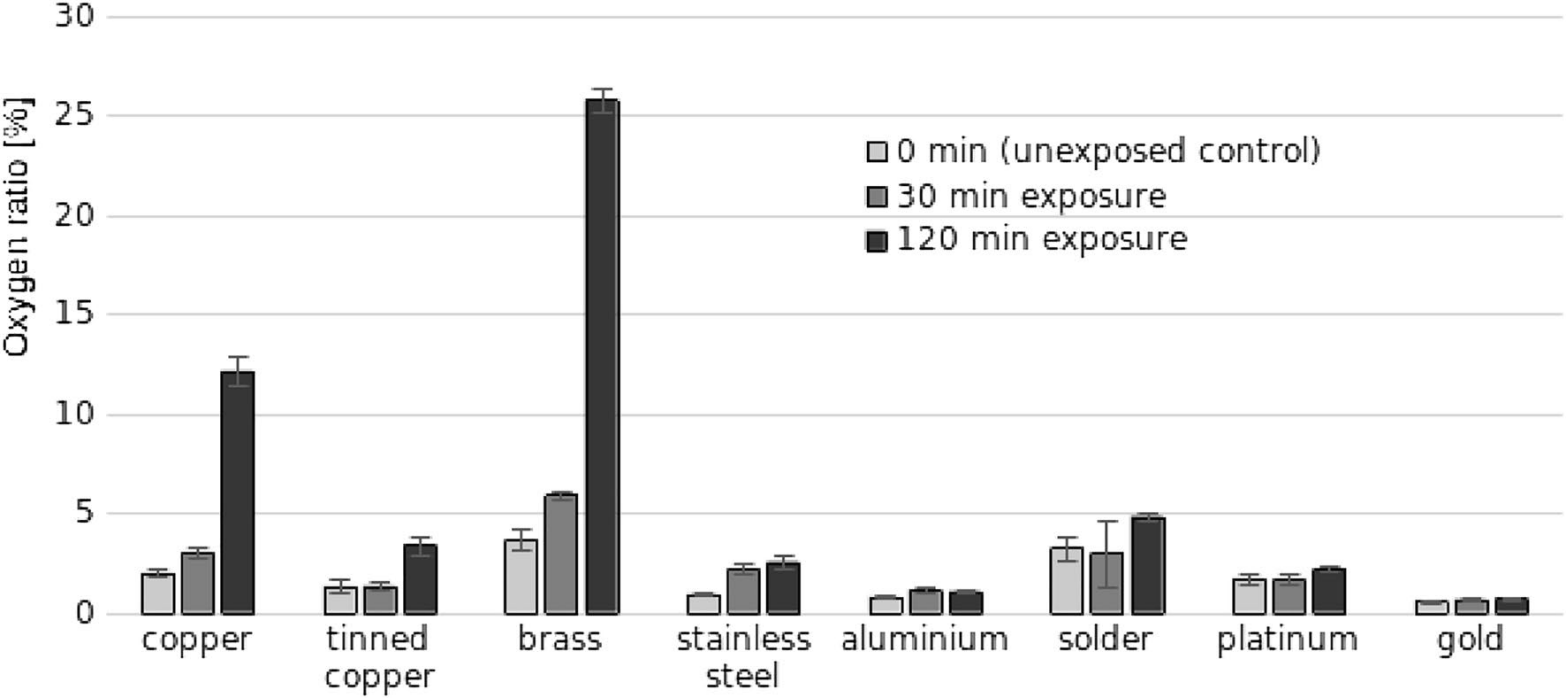
Oxygen mass ratio to other elements in control (0 min exposure) and for 30 and 120 min NTP-exposed metal samples. Results are shown as average of three replicates ± standard deviations.

Changes in oxygen ratio of control (0 min exposure) and NTP-exposed (30 and 120 min) metals determined using EDS are shown in Fig. 3. Detailed measurement of detected elements can be found in Supplementary material (Table S2). The oxygen ratio increased the most, by almost 40% after 120 min of NTP exposure, in brass. While a considerable increase, by more than 20% after 120 min, was observed in copper, tinned copper was only slightly affected, by approximately 7%. Other metals showed only minor or no changes in the measured oxygen ratio.

### Changes in properties of electronic components after exposure to NTP

Electrical properties of control (0 min exposure) and NTP-exposed (30 and 120 min) resistors and capacitors (Table 1) and trimmers showed no significant changes. For trimmers the measured resistance *R*_A_ and *R*_B_ differences are less than 2%.

**Table 1:** Resistance and capacity of unexposed control (0 min) and non-thermal plasma (NTP)-exposed resistors and capacitors.

Component	0 min (control)	30 min NTP exposure	120 min NTP exposure
SMD resistor *R* = 15 Ω	14.99 ± 0.03 Ω	14.98 ± 0.03 Ω	14.99 ± 0.03 Ω
SMD capacitor *R* = 56 nF	58 ± 5 nF	55 ± 5 nF	59 ± 5 nF
THT capacitor *C* = 10 nF	11.1 ± 0.3 nF	11.4 ± 0.3 nF	11.1 ± 0.3 nF
THT capacitor *C* = 15 nF	16.0 ± 0.3 nF	16.2 ± 0.3 nF	15.9 ± 0.3 nF

Average of six replicates ± standard deviations are listed.

*SMD* surface mount device, *THT* through-hole technology.

The particular measured values are shown in the Supplementary material (Table S3).

**Figure 4:**
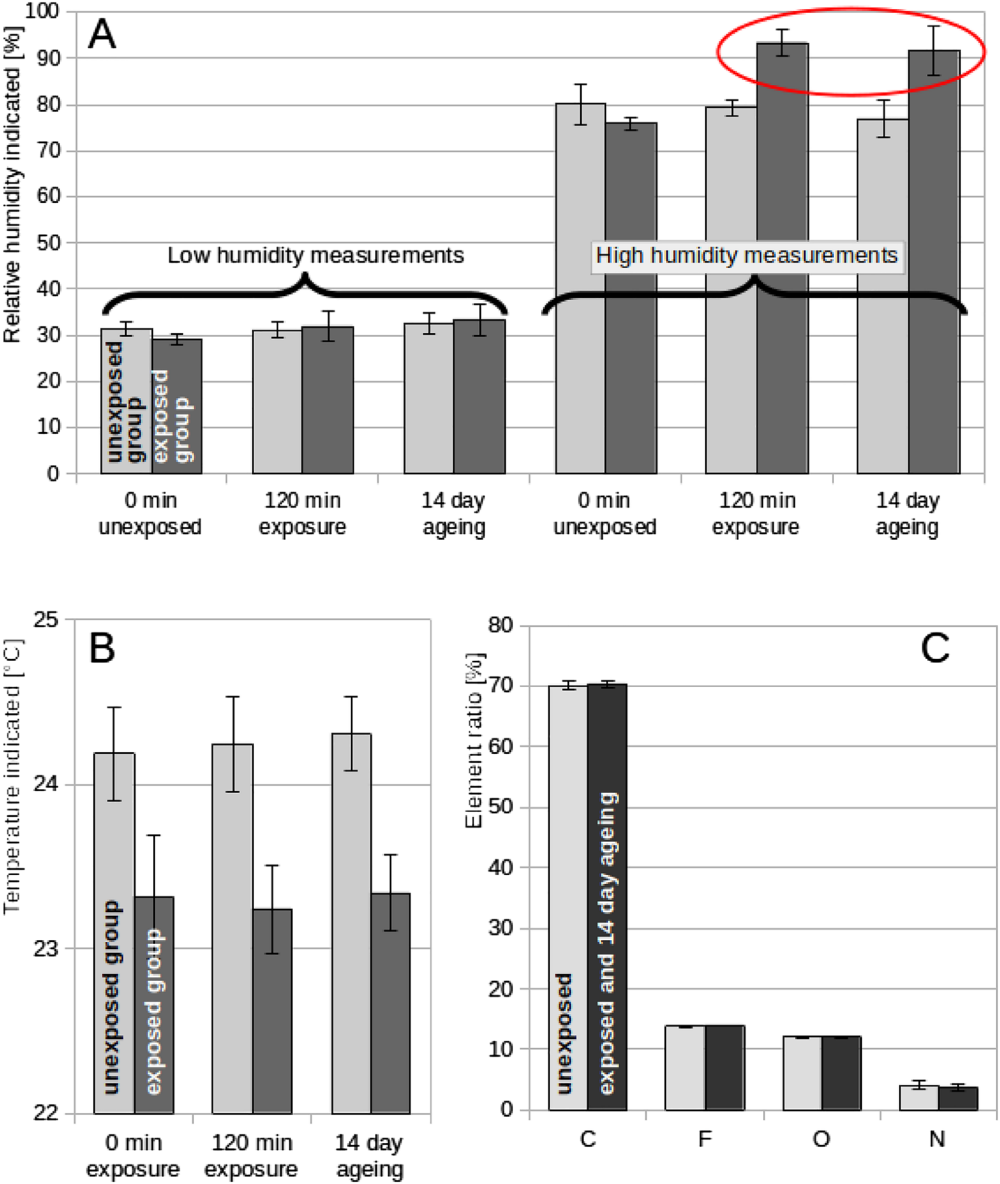
The values indicated by combined humidity and temperature sensors in unexposed (0 min) sensor group and the NTP-exposed group: (**A**) Relative humidity, (**B**) temperature – the red encircle highlights the detected difference. EDS analysis (**C**) of the active layer of unexposed (0 min) humidity sensors and after 120 min exposure and 14 days of ageing. Results are shown as average of six replicates ± standard deviations.

Results obtained for the control (0 min exposure), NTP-exposed (30 and 120 min) and for 14 days aged combined temperature and humidity sensors are presented in Fig. 4. Relative humidity (Fig. 4A) measurements carried out at a low relative humidity (approximately 30%) detected no substantial differences between the control and the NTP-exposed groups. However, difference of approximately 10%, and hence exceeding the declared sensor accuracy, was detected in measurements carried out at high relative humidity (approximately 80%). This increase was observed both directly after exposure to NTP and after 14 days of ageing. Temperature measurements (Fig. 4B) showed a difference of approximately 1 °C between the control and NTP-exposed groups, even though individual sensors were randomly distributed into the groups. However, this difference does not exceed the declared sensor accuracy (± 2 °C) and the values in each group did not change upon NTP treatment. Therefore, no changes in temperature measurements due to NTP were observed. To further address the observed change in humidity measurements, element ratio in the sensor active layer was determined using EDS (Fig. 4C). The elements C, F, O and N were found to be the most abundant. Nevertheless, no changes in their abundance were detected.

## Discussion

This is one of the initial articles that describes the possible risks when decontaminating objects using NTP. For this reason, we exposed selected sensitive materials of daily use (paper, metals, and electric components) to NTP and determined their main parameters after exposure. The determination of the changes in the properties of sensitive materials after exposure to NTP obtained in this study brings expected and new insights. To the best of our knowledge, no such study focusing on undesirable modifications of sensitive materials subjected to NTP treatment for potential decontamination has been published yet and we have shown that although several minor modifications occur, none of the treated materials were destroyed in a way that would make reuse impossible.

In general, NTP generated in the air, which was the subject of this study, is considered a rich source of RNS and ROS that have a strong oxidizing ability. Therefore, when using NTP for the microbial decontamination of sensitive materials, among other things, it is appropriate to determine the degree of potential damage or modification of their surfaces or other properties. As indicated by the results obtained for NTP exposure to paper, its modification can be considered to be a slight damage. A few years ago, we conducted a similar study on disinfection of historical paper archival documents’ disinfection^27^. We concluded that NTP is not suitable for decontamination or disinfection of historical paper documents. Due to their high degree of contamination, a high dose of NTP was needed, and considering the nature of the rare and valuable documents, any damage was undesirable. However, here we demonstrated that the common paper-type modification is only slight even after 120 min of NTP exposure and does not affect its usability. In everyday conditions, a person commonly comes into contact with documents representing by their nature valuables or official documents, money, recipes, etc. and their very long-term archiving is not expected. As was shown, there were no changes in the mechanical properties of the paper. The brightness and whiteness of the papers increased slightly, which can be attributed to the whitening effects of ROS. This is consistent with published studies that describe, for example, teeth whitening with NTP^32^ or the whitening effects of H_2_O_2_, which may also play a role^33^. The specific mechanism of whitening is not easy to determine. Oxidation could not be observed on EDS because the paper itself contains a large amount of oxygen and its slight increase is below the resolution capability. However, it is possible to assume that the whitening may be caused by both the oxidation of molecules that are part of the paper structure. The destruction of large molecules was shown in the reduction of the degree of polymerization of cellulose, i.e., by breaking the long polymer chains. This phenomenon is often associated with the natural ageing process of paper, and therefore the exposure of papers to NTP can be considered as their artificial ageing.

The treatment of the surfaces of selected metals with NTP in our study confirmed the well-known fact that their surface may be oxidized by its action^29^. In the case of copper, tinned copper and brass materials, strong oxidation occurs. Slight oxidation, which may not be significant in general, occurs with stainless steel, and some prompt of oxidation can be seen for platinum. In the case of solder oxidation, the high uncertainty is likely due to the nature of the solder itself; therefore, the oxidation can potentially be hidden. The other metals were not oxidized. Aluminum is usually assumed to have a thin oxide layer, but this effectively prevents further oxidation and protects the metal itself. For gold, no oxidation was even expected^24^. Although copper and brass are oxidized, the action of NTP can be considered gentle and NTP decontamination can be used without risk of damage. The oxidation found takes place only on the surface and can be expected to be much lower compared to chemical disinfectants. Copper is often used as an electrical contact and mechanical abrasion near the socket can be expected. It is important not to disturb the contact solder to prevent the potential formation of a cold solder joint in the electrical contact of electronic components.

The passive SMD and THT electronic components and the resistance trimmer, where the wiper is running on the exposed layer, did not change their value when treated with NTP. This, together with the unconfirmed oxidation of solder serving as the electric contact material, can encourage us to consider that NTP treatment does not impact the electronic devices. However, electronic devices may also active components. In this study, we tested the combined digital temperature and humidity sensor, for which no damage to sensor measurement and communication circuits was observed. Furthermore, there was no change in the indicated temperature, but there was a significant change in the indicated humidity. Humidity measurement is based on balancing the humidity of the surrounding air with the humidity of the sensor's active layer. It is possible to reasonably assume that the application of NTP resulted in the modification of this active layer by increasing its wettability. The change in wettability or surface energy is often interpreted as oxidation of the surface. However, our study did not confirm the increased presence of oxygen or other elements in the active layer of the sensor. Similar effects of NTP have been documented previously^34–36^. In the last mentioned also the effect of ageing is reported, where the wettability increases strongly after exposure to NTP but in several days the surface consecutively relaxes toward original values. It is interesting that for our sensor, this shift is of a long-term nature and no backward relaxation of the layer was observed even after 14 days of ageing. An explanation for this phenomenon is, however, not available at this time. Although these results sound very optimistic, it would be useful to include also other materials in the study and to look at multiple treatments of objects or long-term studies to gain a deeper understanding of the processes involved in NTP decontamination. However, from the stated results and the possible reasons, it can be concluded that NTP can certainly be used for the decontamination or disinfection of objects without significant damage to them, even of sensitive materials. Nevertheless , increased attention must be paid to the decontamination of active sensitive layers, which needs to be studied in more detail.

## Conclusion

Paper, several metal samples, and passive and active electronic components were treated with NTP and their possible damage was evaluated. Only slight changes in the characteristics of the paper were observed, such as a whitening of the paper and a decrease in the pH value and degree of polymerization. For metal samples, slight oxidation was observed for copper and tinned copper, brass, and very mild oxidation was also observed for stainless steel. On the other hand, for aluminum, gold, and platinum, no modification was confirmed. No changes were observed for passive electronic components; however, the values indicated by the active digital humidity sensor increased after NTP treatment of the sensor, whereas for the temperature sensor the indicated values remain unchanged. In general, it is possible to use NTP for decontamination and decontamination of sensitive materials without the risk of damage.

### Supplementary Information

Supplementary Tables.

## Data Availability

The datasets used and/or analyzed during the current study are available from the corresponding author on reasonable request.
