# Supplementary material for: Non-thermal plasma disinfecting procedure is harmless to delicate items of everyday use
Source: Sci Rep. 2023 Sep 19;13:15479. doi: 10.1038/s41598-023-42405-6 (PMC10509187; doi:10.1038/s41598-023-42405-6)
Supplement: Supplementary file 1 — Supplementary Tables. [file 41598_2023_42405_MOESM1_ESM.docx]

Table S1. Mechanical parameters of paper samples after exposure to NTP. The paper samples were cut in both directions of their machine direction (MD) and cross direction (CD). Results are shown as averages of six repetitions ± standard deviation.

|  | **NTP exposure [min]** | | | | | | | | |
| --- | --- | --- | --- | --- | --- | --- | --- | --- | --- |
|  | **0 (control)** | | | **30** | | | **120** | | |
| Thickness [mm] | 0.187 | ± | 0.05 | 0.186 | ± | 0.05 | 0.190 | ± | 0.05 |
| Grammage [g/m^2^] | 87.3 | ± | 0.30 | 87.8 | ± | 1.30 | 88.3 | ± | 0.90 |
| **Machine direction (MD)** |  |  |  |  |  |  |  |  |  |
| Tensile strength [kN/m] | 2.33 | ± | 0.05 | 2.30 | ± | 0.07 | 2.33 | ± | 0.12 |
| Breaking strength [MPa] | 12.75 | ± | 0.14 | 12.83 | ± | 0.26 | 12.76 | ± | 0.33 |
| Breaking length [km] | 2.72 | ± | 0.05 | 2.67 | ± | 0.08 | 2.68 | ± | 0.14 |
| Relative elongation [%] | 1.60 | ± | 0.06 | 1.60 | ± | 0.11 | 1.60 | ± | 0.19 |
| **Cross direction (CD)** |  |  |  |  |  |  |  |  |  |
| Tensile strength [kN/m] | 1.52 | ± | 0.03 | 1.52 | ± | 0.06 | 1.53 | ± | 0.05 |
| Breaking strength [MPa] | 8.80 | ± | 0.07 | 8.90 | ± | 0.35 | 8.67 | ± | 0.16 |
| Breaking length [km] | 1.77 | ± | 0.03 | 1.77 | ± | 0.07 | 1.76 | ± | 0.06 |
| Relative elongation [%] | 2.60 | ± | 0.15 | 2.60 | ± | 0.35 | 2.50 | ± | 0.14 |

Table S2. Measured values of quantities for electric components.

|  | 0 min (control) | 30 min NTP exposure | 120 min NTP exposure |
| --- | --- | --- | --- |
| **SMD resistors 15 Ohm** | 15.00±0.03 Ohm | 14.99±0.04 Ohm | 15.00±0.03 Ohm |
| **SMD capacitors 56 nF** | 59±10 nF | 55±6 nF | 59±10 nF |
| **THT capacitors 10 nF** | 11.2±0.3 nF | 11.4±0.3 nF | 11.1±0.3 nF |
| **THT capacitors 15 nF** | 16.0±0.3 nF | 16.2±0.3 nF | 15.9±0.3 nF |
|  |  |  |  |
| **Trimmers 10 kOhm** | first terminal and the wiper *R*_a_ / kOhm | | |
| 1 | 4.83 | 4.84 | 4.85 |
| 2 | 7.38 | 7.37 | 7.37 |
| 3 | 6.37 | 6.35 | 6.34 |
| 4 | 6.07 | 6.07 | 6.07 |
| 5 | 7.05 | 7.05 | 7.05 |
| 6 | 6.04 | 6.05 | 6.05 |
|  | wiper and the second terminal *R*_b_ / kOhm | | |
| 1 | 4.17 | 4.17 | 4.18 |
| 2 | 4.2 | 4.18 | 4.18 |
| 3 | 4.66 | 4.68 | 4.68 |
| 4 | 3.91 | 3.91 | 3.91 |
| 5 | 4.33 | 4.33 | 4.34 |
| 6 | 4.21 | 4.21 | 4.21 |

Table S3. EDS mass percentace of elements for metalic samples. Results are shown as averages of three repetitions ± standard deviation.

| **Copper** |  |  |  |  |  |  |  |
| --- | --- | --- | --- | --- | --- | --- | --- |
| analyzed element | Copper | Carbon | Oxygen | Tin | Nitrogen |  |  |
| **0 min (control)** | 91.3±0.5 | 6.7±0.2 | 2.0±0.3 | 0.0±0.1 | 0.0±0.1 |  |  |
| **30 min NTP exposure** | 90.9±0.5 | 6.1±0.3 | 2.8±0.2 | 0.0±0.1 | 0.0±0.1 |  |  |
| **120 min NTP exposure** | 79.9±2.9 | 6.1±1.6 | 12.1±0.8 | 0.7±0.5 | 1.4±1.5 |  |  |
|  |  |  |  |  |  |  |  |
| **Tinned copper** |  |  |  |  |  |  |  |
| analyzed element | Tin | Copper | Carbon | Nitrogen | Lead | Oxygen | Aluminium |
| **0 min (control)** | 69.8±0.8 | 21.1±1.6 | 4.2±0.7 | 2.6±0.4 | 0.8±0.2 | 1.3±0.4 | 0.2±0.3 |
| **30 min NTP exposure** | 75.4±3.3 | 16.3±2.6 | 3.9±0.6 | 3.1±0.8 | 0.0±0.1 | 1.3±0.2 | 0.0±0.1 |
| **120 min NTP exposure** | 45.4±34.4 | 17±10 | 3.7±2.9 | 2.3±1.5 | 0.5±0.3 | 2.3±1.7 | 0.0±0.1 |
|  |  |  |  |  |  |  |  |
| **Brass** |  |  |  |  |  |  |  |
| analyzed element | Copper | Carbon | Zinc | Oxygen | Nitrogen |  |  |
| **0 min (control)** | 59.0±1.0 | 8.7±1.3 | 28.6±0.2 | 3.7±0.5 | 0.0±0.1 |  |  |
| **30 min NTP exposure** | 58.9±0.7 | 6.3±1.8 | 27.7±0.2 | 5.9±0.2 | 1.2±1.1 |  |  |
| **120 min NTP exposure** | 44.5±0.7 | 2.9±0.9 | 21.8±0.4 | 25.8±0.6 | 5.0±0.2 |  |  |
|  |  |  |  |  |  |  |  |
| **Stainless steel** |  |  |  |  |  |  |  |
| analyzed element | Carbon | Oxygen | Silicon | Chromium | Manganese | Iron | Nickel |
| **0 min (control)** | 4.0±0.5 | 1.0±0.1 | 0.6±0.1 | 17.6±0.1 | 0.7±0.1 | 68.3±0.4 | 7.7±0.2 |
| **30 min NTP exposure** | 7.2±1.2 | 2.2±0.3 | 2.2±0.1 | 16.6±0.3 | 0.6±0.1 | 63.8±1.2 | 7.3±0.1 |
| **120 min NTP exposure** | 8.3±2.0 | 2.5±0.3 | 2.5±0.1 | 16.3±0.3 | 0.7±0.1 | 62.5±2.0 | 7.3±0.2 |
|  |  |  |  |  |  |  |  |
| **Aluminium** |  |  |  |  |  |  |  |
| analyzed element | Aluminium | Carbon | Oxygen |  |  |  |  |
| **0 min (control)** | 96.6±0.5 | 2.6±0.4 | 0.8±0.1 |  |  |  |  |
| **30 min NTP exposure** | 95.5±0.2 | 3.4±0.1 | 1.1±0.2 |  |  |  |  |
| **120 min NTP exposure** | 95.2±0.7 | 3.7±0.7 | 1.1±0.1 |  |  |  |  |
|  |  |  |  |  |  |  |  |
| **Solder** |  |  |  |  |  |  |  |
| analyzed element | Lead | Carbon | Tin | Oxygen | Nitrogen |  |  |
| **0 min (control)** | 64.5±3.9 | 3.2±0.2 | 28.2±1.9 | 3.2±0.6 | 0.9±1.6 |  |  |
| **30 min NTP exposure** | 46.5±12.7 | 3.2±0.6 | 54.1±14.6 | 3.0±1.6 | 3.7±0.7 |  |  |
| **120 min NTP exposure** | 66.4±6.2 | 5.1±0.6 | 21.6±5.1 | 4.8±0.2 | 2.1±2.0 |  |  |
|  |  |  |  |  |  |  |  |
| **Platinum** |  |  |  |  |  |  |  |
| analyzed element | Platinum | Carbon | Oxygen |  |  |  |  |
| **0 min (control)** | 87.7±2.5 | 10.6±2.2 | 1.7±0.3 |  |  |  |  |
| **30 min NTP exposure** | 90.3±0.5 | 8.1±0.7 | 1.7±0.3 |  |  |  |  |
| **120 min NTP exposure** | 88.8±0.8 | 8.9±0.9 | 2.2±0.1 |  |  |  |  |
|  |  |  |  |  |  |  |  |
| **Gold** |  |  |  |  |  |  |  |
| analyzed element | Gold | Carbon | Oxygen |  |  |  |  |
| **0 min (control)** | 95.3±0.3 | 4.1±0.2 | 0.6±0.1 |  |  |  |  |
| **30 min NTP exposure** | 95.5±0.4 | 3.8±0.3 | 0.7±0.1 |  |  |  |  |
| **120 min NTP exposure** | 95.8±0.7 | 3.6±0.6 | 0.7±0.1 |  |  |  |  |
